# Bariatric surgery for metabolic unhealthy obesity (MUO) during the COVID era: short-term results of a high-volume center

**DOI:** 10.1007/s40519-022-01438-y

**Published:** 2022-07-19

**Authors:** Valeria Guglielmi, Michela Campanelli, Emanuela Bianciardi, Domenico Benavoli, Luca Colangeli, Monica D’Adamo, Paolo Sbraccia, Paolo Gentileschi

**Affiliations:** 1 Department of Systems Medicine, University of Rome Tor Vergata, Via Montpellier 1, 00133 Rome, Italy; 2 Internal Medicine Unit and Obesity Center, University Hospital Policlinico Tor Vergata, Rome, Italy; 3 Department of Bariatric and Metabolic Surgery, San Carlo of Nancy Hospital, University of Rome Tor Vergata, Rome, Italy; 4 Chair of Psychiatry, Department of Systems Medicine, University of Rome Tor Vergata, Rome, Italy

**Keywords:** Laparoscopic bariatric surgery, COVID-19, Metabolically unhealthy obese, Sleeve gastrectomy, Weight loss

## Abstract

**Purpose:**

During the coronavirus disease 19 (COVID-19) outbreak, most public hospitals worldwide have been forced to postpone a major part of bariatric surgery (BS) operations with unfavorable consequences for weight and obesity complications. The aim of this study was to evaluate the effectiveness and safety of laparoscopic BS on subjects with metabolically unhealthy obesity (MUO) during COVID-19 pandemic in a high-volume Italian center.

**Methods:**

Between March 2020 and January 2021, all patients with MUO submitted to laparoscopic BS (sleeve gastrectomy [SG], one anastomosis gastric bypass [OAGB] and Roux-en-Y gastric bypass [RYGB]) were enrolled according to the ATP III Guidelines, with a minimum follow-up of 3 months.

**Results:**

In the study period, 210 patients with MUO underwent laparoscopic BS (77 RYGB, 85 SG and 48 OAGB) in our obesity referral center. Postoperative major complications occurred in 4 patients (1.9%) with zero mortality. At 9-month follow-up, a total weight loss (TWL) of 28.2 ± 18.4, 26.1 ± 23.1 and 24.5 ± 11.3% (*p* = 0.042) was observed in RYGB, OAGB and SG groups, respectively. The rate of comorbidity resolution was very similar for all type of surgeries (*p* = 0.871). Only two cases of postoperative SARS-CoV-2 infection were registered (0.9%) and both cases resolved with medical therapy and observation.

**Conclusion:**

Among the patients studied, all surgical techniques were safe and effective for MUO during the COVID era. This group of patients is at high risk for general and SARS-CoV-2-related mortality and therefore should be prioritized for BS.

**Level of evidence:**

Level III, single-center retrospective cohort study.

## Introduction

Coronavirus disease 2019 (COVID-19), first identified in December 2019 in Wuhan, China, is an infectious disease caused by severe acute respiratory syndrome coronavirus 2 (SARS-CoV-2). Since then, SARS‐CoV‐2 has spread throughout the world, leading the World Health Organization (WHO) to issue a pandemic alert on 11 March 2020. On March 2022, the total number of confirmed cases reached over 440 million with more than 5.9 million deaths worldwide [1].

According to COVID-19 surveillance reports of European Centre for Disease Prevention and Control (ECDC) and Centers for Disease Control and Prevention (CDC) from US, some underlying medical conditions including obesity as well as hypertension, diabetes, cardiovascular disease, chronic respiratory disease, chronic kidney disease, immune compromised status, cancer, and smoking are associated with worse COVID-19 outcomes [2].

Therefore, it is not surprising that especially metabolically unhealthy obesity (MUO), a subset of obesity characterized by cardiometabolic complications, higher liver fat content, insulin resistance, inflammation and adipose tissue dysfunction, has been recognized as a major risk factor for COVID-19 severity and mortality [3–7].

Robust data have indicated bariatric surgery (BS) as a valid option for significant weight loss and remission of comorbidities in a weight-dependent and independent manner in patients with MUO [8–10]. Nevertheless, during the COVID-19 outbreak, most public hospitals worldwide have been obliged to postpone a major part of elective operations including BS to increase inpatient capacity, to reconvert postoperative intensive care units to intensive care for COVID-19 critical patients and to minimize intraoperative risks for viral contagion [11]. Among the reasons why most bariatric elective interventions were suspended during the most acute phase of COVID-19 pandemic, there is also the reduced access to non-urgent outpatient care which might constrain the postoperative monitoring for potential surgical and nutritional complications.

As soon as COVID-19 pandemic passed its first peak, questions raised about the strategies to adopt for metabolic and weight control in patients awaiting BS. Indeed, deferring the treatment of obesity, especially under lockdown conditions which entail lifestyle adjustments, mobility restrictions and sleep disruption, may have caused further weight gain and the worsening of obesity complications [11, 12]. In our experience, the lockdown had a negative impact on patients’ psychological well-being and eating habits [13]. Most patients and caregivers reported anxiety, depression and poor quality of life, which deteriorated with increasing wait time, especially in women and in those of younger age and lower socio-economic status [14].

Also, recommendations to safely reintroduce BS [15] and to help prioritize patients at the greatest risk of harm from delayed treatment or that most benefit from BS in COVID-19 era have been issued [16, 17].

However, little is known about the real impact of COVID-19 pandemic on the effectiveness and safety of laparoscopic BS during COVID-19 pandemic in high-risk patients with obesity if we exclude a small series of asymptomatic COVID-19-positive patients undergoing BS during the initial phase of outbreak in Iran [18] and short-term (over 30 postoperative days) assessment of COVID-19-related morbidity/mortality after BS during the phase 2–3 of pandemic [19, 20].

Herein, we present the results, in terms of efficacy and safety, of laparoscopic bariatric operations in patients with MUO performed during COVID-19 pandemic, in a high-volume Italian center after the curve of new COVID-19 cases had flattened.

## Methods and procedures

Between March 2020 and January 2021, 210 patients undergoing laparoscopic BS (sleeve gastrectomy [SG], one anastomosis gastric bypass [OAGB] and Roux-en-Y gastric bypass [RYGB]) were enrolled. Patients were eligible for inclusion if they were affected by MUO, were older than 18 years and if they were followed up for at least 3 months after BS. MUO was defined as having > 3 of the following metabolic syndrome components: high systolic and diastolic blood pressure, high serum triglycerides [21], low HDL-Cholesterol [HDL-C] and high fasting blood glucose levels [22, 23]. All patients underwent a multidisciplinary evaluation according to a standardized clinical protocol and were consequently assigned to surgical treatment according to European criteria [24]. Preoperative work-up included esophagogastroscopy, barium swallow, blood samples, chest X-ray, electrocardiogram and, if needed, spirometry, echocardiography and polysomnography. Before entry the hospital, all patients were screened for SARS-CoV-2 infection by a questionnaire and nasopharyngeal swab. In line with international and national guidelines [16, 25], 2–3 days before admission, the patients were interviewed by telephone by using a standardized questionnaire [26]. Twenty-four hours before hospitalization, the hospital medical staff repeated the interview and swab the patient. Demographics, comorbidities, anthropometric and clinical parameters and data on surgical procedure, complications and reoperations were prospectively collected. Clinical and anthropometric evaluation and biochemical testing (glucose and lipid profile and liver enzymes) were assessed at baseline (mean 20 ± 7 days prior to surgery) and 1, 3, 6, 9, 12 months after surgery. All surgical procedures were performed by the same team using standard techniques.

## Statistical analysis

Quantitative variables that followed a normal distribution were summarized as means ± standard deviations (SD). Medians and ranges were recorded for non-Gaussian variables. Qualitative variables were summarized by number and as percentage of cases. Comparisons between groups were made using Student’s unpaired *t* test and analysis of variance (ANOVA) with Bonferroni post hoc analysis. A comparison of qualitative variables was performed by Chi-square test. *P* value < 0.05 based on two-sided test was considered statistically significant. Statistical analysis was performed by using SPSS 19.0 software (SPSS, Chicago).

## Results

Among the 210 MUO patients undergoing laparoscopic BS, 135 (6429%) were females and 75 (358%) males, 77 underwent RYGB, 48 OAGB, and 85 SG.

Preoperative characteristics of patients for each group are summarized in Table 1.

**Table 1:** Preoperative patients’ characteristics for each group of surgery

	RYGB (*n* = 77)	OAGB (*n* = 48)	SG (*n* = 85)	*P *value
BMI (Kg/m^2^)	46.4 ± 7.2	43.5 ± 9.2	44.4 ± 6.9	0.09
Waist circumference				
> 102 cm (Males)	38 (49.5%)	19 (39%)	44 (52%)	0.06
> 88 cm (Females)	41 (53%)	22 (45%)	32 (38%)	0.06
Mean waist circumference (cm)	118.7 ± 32.1	114.7 ± 23.4	116.6 ± 17.4	0.11
HP or use of HP therapies	55 (65%)	27 (57%)	47 (55%)	0.08
HDL-C < 40 mg/dl (males) < 50 mg/dl (females)	68 (89%)	32 (67%)	61 (72%)	0.07
Mean HDL-C levels (mg/dl)	39.7 ± 11	37.6 ± 12.1	39.6 ± 12.3	0.12
TG (> 150 mg/dl)	52 (67%)	27 (56%)	58 (68%)	0.06
Mean TG levels (mg/dl)	154.5 ± 72.6	153.9 ± 60.7	150 ± 72.8	0.11
Pre-op T2D	13 (16.3%)	7 (15.3%)	5 (5.4%)	0.06
Mean fasting blood glucose levels (mg/dl)	118.7 ± 22.1	124.7 ± 23.4	126.6 ± 13.4	0.09

Abbreviations: *HDL-C* HDL cholesterol, *HP* Hypertension, *OAGB* One-anastomosis gastric bypass, *RYGB* Roux-en-Y gastric bypass, *SG* Sleeve gastrectomy, *T2D* Type 2 diabetes, *TG* Triglycerides

Preoperative SARS-CoV-2 positive swabs were detected in eight patients (3.8%) who were delayed for surgery until they convert to negative and, as long-term effects or complications of COVID-19 are described, after multidisciplinary team approval. In the meanwhile, these patients were advised how to mitigate harm from delaying surgery.

With regard to the postoperative results, no major intraoperative complications were recorded and neither intraoperative nor perioperative deaths within 24 h after surgery occurred. As shown in Table 2, postoperative (at a mean 9-month follow-up) major complications were recorded in four patients (1.9%). Of these, three were due to staple line leaks in patients of SG group and occurred 7, 9 and 11 days after surgery. In all cases, the leaks were located at the gastroesophageal junction area, along the staple line, and were treated with laparoscopic drainage, followed by endoscopic gastroesophageal stenting. One patient who had undergone RYGB had an intra-abdominal abscess 3 weeks after surgery and underwent CT-guided percutaneous drainage. Minor complications were observed in five patients (2.4%). Of these, one case of port-site incisional hernia after 3 months after SG was detected and three cases of subcutaneous infection medically treated after RYGB occurred; eventually, in one patient undergone OAGB, a subcutaneous hematoma was diagnosed 3 weeks after surgery and treated with percutaneous drainage. After a mean follow-up of 9 months, a greater total weight loss (TWL) was observed in the RYGB group compared to SG and OAGB. The comorbidities’ resolution rate was similar for all the procedures (*p* = 0.871) (Table 2).

**Table 2:** Short-term results after bariatric surgery

	RYGB	OAGB	SG	*P* value
No. of patient	77 (36.7%)	48 (22.8%)	85 (40.5%)	0.072
Major complications	1 (1.3%)	–	3 (3.5%)	0.04
Minor complications	3 (3.9%)	1 (2.1%)	1 (1.2%)	0.07
Excess weight loss (%)	76.2 ± 21.4	71.1 ± 23.1	66.4 ± 19.1	0.031
Total weight loss (%)	28.2 ± 18.4	26.1 ± 23.1	24.5 ± 11.3	0.042
BMI (Kg/m^2^)	32.4 ± 8.3	33.5 ± 7.9	34.6 ± 7.1	0.061
Fasting blood glucose (mg/dl)	98.7 ± 32.5	94.7 ± 33.5	96.6 ± 18.9	0.021

The data reported refer to a mean 9-month follow-up

Abbreviations: *OAGB* One-anastomosis gastric bypass, *RYGB* Roux-en-Y gastric bypass, *SG* Sleeve gastrectomy

In our center, nasal swabs were carried out at discharge if the hospitalization exceeded 48 h. Postoperative SARS-CoV-2 infection was detected in two patients (0.9%) who recovered well after 3 weeks of medical therapy, none of them requiring hospitalization. The first patient, 6 days after surgery, had a nasal swab due to respiratory symptoms and tested positive, and the second one was tested with nasal swab 9 days after surgery for respiratory symptoms and fever onset.

## Discussion

Since the first case of SARS-CoV-2 infections was isolated, COVID-19 pandemic escalated rapidly, so that the unexpected overflow of COVID-19 patients represented an enormous challenge for hospitals and institutions of care and a deep reorganization of national health systems became necessary. In this context, most elective surgery was postponed by the end of February 2020 to increase inpatient capacity and acute care [27]. Bariatric and metabolic procedures were delayed worldwide during the pandemic also to limit the risks related to inadequate postoperative monitoring for surgical and nutritional complications as a consequence of the constrained access to non-urgent outpatient care [16], as well as to minimize the risk of viral contagion during hospitalization. This delay had a negative impact on patients with severe obesity [11, 12, 28, 29], also considering that they are at increased risk for unfavorable COVID-19 outcomes. Indeed, in a series of multivariable‐adjusted analyses based on COVID‐19 patient cohorts, disease severity and mortality were associated not only with older age and male gender [30], but also with several pre-existing medical conditions including obesity, diabetes, hypertension, heart failure and ischemic heart disease among others [2]. In some reports, obesity was even the most associated comorbidity accounting for the majority of COVID-19-related ICU admissions [6, 31], especially in younger patients [32]. Thus, it is not surprising that primarily patients affected by MUO, an obesity phenotype exhibiting adverse cardiometabolic consequences of excess body fat, were at even greater risk of COVID-19-related unfavorable outcomes [7, 33]. In this context, MUO patients were particularly harmed not only by COVID-19 confinement that caused significant lifestyle disruption and worsened their metabolic abnormalities [34], but also by the BS delay.

As soon as COVID-19 pandemic passed its first peak, questions raised about the strategies to reintroduce safely BS [15, 35] and about patients to prioritize [16, 17]. Even though lower risk patients are less likely to have a longer length of hospital stay, readmission or complications [15], most authors recommended prioritizing patients with obesity in more urgent need and with the greatest risk of harm from delayed treatment [16, 17]. These patients are also those who appear to benefit most from BS [9, 36–39].

The primary aim of our retrospective study was to evaluate the impact of performing surgical laparoscopic procedures on a bariatric population without specific preventive measures other than filling out questionnaires on clinical symptoms before admission, nasopharyngeal swabs, keeping inpatient COVID-19/noCOVID-19 pathways separated, social distancing and surgical masks.

Asymptomatic SARS-CoV-2 infections were detected preoperatively by positive nasal/oro-pharyngeal swab (molecular tests) in eight patients (3.8%) who were delayed for surgery, while only two cases of postoperative SARS-CoV-2 infection were registered (0.9%). Both cases resolved with medical therapy and made a full recovery with a negative swab. These results are in line with the incidence of postoperative COVID-19 infections (0.6%) in a cohort of 840 BS patients, although over a much shorter period (2 months) [26].

In our cohort of patients, we recorded the same rate of postoperative complications as the previous year. Namely, postoperative major and minor complications were reported in four (1.9%) and five patients (2.4%), respectively. In 2019, out of the 342 patients undergoing BS, seven developed postoperative complications. In the OAGB group, two patients suffered from late minor complications: one experienced bile reflux (grade I) and one reported vomiting/food intolerance (grade I). Surgical reintervention was needed in one patient for postoperative bleeding from the port insertion site (Clavien–Dindo IIIb). In the SG group, three major complications occurred in the postoperative period (Clavien–Dindo IIIb): 2 gastric leaks and one major bleeding, treated with laparoscopic drainage. One patient who had undergone RYGB had subcutaneous infection medically treated (grade I).

Similarly, bariatric [26], oncologic and emergency [40] patients operated in Northern Italy during the outbreak in March and April 2020 did not experience higher rates of postoperative complications and mortality compared to patients operated the previous year. These data suggest that the afore-mentioned screening of patients before hospital admission and inpatient unspecific preventive measures represent a safe approach.

The RYGB yielded a greater weight loss effect, followed by OABG and SG, with mean %EWL values in line with reports preceding COVID-19 outbreak [10, 41], suggesting that the concomitant COVID-19 pandemic and lockdown did not negatively impact on BS results in terms of body weight loss.

We acknowledged, as the main limitations of the study, the monocentric design and the short postoperative follow-up, even if the rate of patients lost was extremely low (0.48%). However, in keeping with our results, although prioritization of metabolic surgery remains controversial [16], we believe that high-volume centers must be engaged in planning a protocol to ensure their surgical activity in case of other outbreaks, in order to guarantee the safety of patients with severe obesity but at the same time their need for BS, which overlaps the need for reducing their vulnerability to COVID-19.

## What is already known on this subject?

Metabolically unhealthy obesity (MUO), a subset of obesity characterized by cardiometabolic complications, higher liver fat content, insulin resistance, inflammation and adipose tissue dysfunction, has been recognized as a major risk factor for COVID-19 severity and mortality. Bariatric surgery (BS) is a valid option for significant weight loss and remission of comorbidities in a weight-dependent and independent manner in patients with MUO, but during the COVID-19 outbreak, most public hospitals worldwide have been forced to postpone a major part of BS operations with unfavorable consequences for weight and obesity complications.

## What this study adds?

In this study, we present the results of laparoscopic BS on 210 patients from a high-volume Italian center during the COVID-19 pandemic. BS efficacy was not influenced by the pandemic in terms of body weight loss, with mean percentage of excess weight loss values in line with previous reports. Moreover, we recorded the same rate of post-operative complications as the previous year 2019. It would be useful for high-volume centers to plan a protocol to guarantee surgical activity in case of other outbreaks, respecting patients’ safety but at the same time the need for severe obesity treatment.

## Data Availability

All data generated or analyzed during this study are included in this published article.
